# The impact of COVID-19 on health systems, mental health and the potential for nursing

**DOI:** 10.1017/ipm.2020.105

**Published:** 2020-09-16

**Authors:** T. Frawley, F. van Gelderen, S. Somanadhan, K. Coveney, A. Phelan, P. Lynam-Loane, A. De Brún

**Affiliations:** 1 UCD School of Nursing, Midwifery and Health Systems, Belfield, Dublin, Ireland; 2 TCD School of Nursing and Midwifery, 24 D’Olier Street, Dublin, Ireland; 3 Dublin North City Mental Health Services, Phoenix Care Centre, Grangegorman, Dublin

**Keywords:** COVID-19, health systems, person and public involvement, psychiatric – mental health nursing

## Abstract

This paper offers a perspective on nursing and lived experience responses to the COVID-19 pandemic. It charts health systems and mental health impacts with a particular focus on children and adolescents, older people and people availing of mental health services. Issues of moral distress and the nursing reaction are considered alongside psychological and social concerns which continue to rapidly evolve. The perspective of a person attending adult community mental health services and the experience of engaging with a mental health service remotely is provided. Matters of note for acute inpatient mental health nursing are highlighted and informed by the lived experience of a mental health nurse. The need for integrated health systems responses across nursing disciplines and the wider interdisciplinary team is elucidated.

## Introduction

Coronavirus (COVID-19) has emerged as the first global pandemic in the 21st century (Anderson *et al.* 2020). COVID-19 is an infection from the new coronavirus severe acute respiratory syndrome COVID-2 (SARS-CoV-2). Historically, coronaviruses only caused infections in animals. To date, we know of at least seven that have transferred to human beings. Most of these corona-related infections have caused mild symptoms. However, two, the Middle Eastern respiratory syndrome (MERS) and SARS have proved to be more severe and resulted in deaths (Ludwig & Zarbock, 2020). Political and health systems’ responses to COVID-19 have demanded extraordinary public health measures at a population level to respond to modifiable factors such as opportunity and transmission (Kucharski, 2020). Globally, the COVID-19 pandemic has imposed significant restrictions on social contact. In Ireland, a series of targeted social movement measures were implemented from 27th March 2020. These included practices such as social distancing and cocooning for people over 70 years and those with various morbidities. Responses have also been focused on mass population based testing, isolation of those who are positive, contact tracing and social isolation of contacts. While the mental health impacts of this pandemic are not fully clarified, it is reasonable to project that there will be unintended, yet enduring impacts in the context of individual mental health, population health as well as impacts on the way health services are delivered in the medium term until a vaccine is isolated.

The vast majority of COVID-19-related publications have centred on the physical aspects and consequences of the viral infection. This paper provides an account of potential mental health impacts of the pandemic on health systems, specifically related to healthcare workers’ moral distress and a consideration of two population groups (children and older people). We also consider the effects on individuals within society as well as providing an insider perspective using two informants, namely, a person availing of and a person employed in mental health services. These foci enabled a preliminary exploration of the context of adapting to new ways of receiving care and working within the limitations of the COVID-19 pandemic (Anderson *et al.* 2020). Although the dominant health system concern has been on addressing the immediacy of the physical impact of COVID-19, mental health aspects resulting from psychological pressure are equally important and will require appropriate responses both for staff and vulnerable groups. The paper concludes with a discussion of the role of mental health nursing as a key resource within an integrated response to COVID-19.

## Health systems and moral distress

While global experts have long predicted a pandemic of the scale and scope experienced as a result of COVID-19, health systems have differed in their responses and perspectives particularly in the context of the level of preparedness, management and monitoring (Davidson & Szanton, 2020). For example, initial issues pertained to significant challenges and mobility in deploying the necessary resources and equipment [e.g. personal protective equipment, (PPE)] required to contain the spread of the virus. Internationally, a matter of concern is the high proportion of healthcare staff who become ill through COVID-19 themselves with Black, Asian and minority ethnic groups (BAME) appearing particularly vulnerable (Chakravorty *et al*. 2020; Lacobussi, 2020).

By 9th May, a total of 6,669 healthcare professionals were confirmed as COVID-19 positive in Ireland (Power, 2020), representing a significant proportion of the total number of recorded cases [Health Protection Surveillance Centre (HPSC, 2020)]. In addition, the Irish Nurses and Midwives Organisation (INMO) has noted that as of mid-April, 10% of all diagnosed COVID-19 infections in Ireland were nurses (INMO, 2020). Reducing the incidence of nurses contracting COVID-19 is vital not only from a personal health perspective but also to ensure that appropriate staffing levels in services can be maintained. This is a challenge, especially in the major urban centres, where there is a higher infection rate (HPSC, 2020). Similar to many areas within the health system, mental health nursing has experienced a staffing shortage (Frawley *et al*. 2018; Health Service Executive, 2018). While other care environments, such as nursing homes, have experienced a disproportionate impact in terms of clusters and death rates (HPSC, 2020), it is also of concern that as of Friday, 8th May, 16 residents with COVID-19 had died in mental health services while the Mental Health Commission described delays in staff testing as unacceptable (Cullen, 2020).

Given the increasing demands on an already stretched healthcare workforce, it is crucial to understand and address the psychological burden on staff. These efforts must seek to alleviate principal sources of anxiety among healthcare staff currently. Some of these concerns relate to access to PPE, balancing one’s own mental and physical health with patient care, fear of exposing family members to the virus, supporting other family members, having access to childcare, fear of developing symptoms and increased working demands (Shanafelt *et al*. 2020). Additionally, staff are at a higher risk of burnout and of emotional and physical exhaustion. Moral injury, or the ‘psychological distress that results from actions, or the lack of them, which violate someone’s moral or ethical code’, has been highlighted as a particular risk for healthcare staff at this time (Greenberg *et al*. 2020:1) and could contribute to the development of mental health difficulties among staff (Williamson *et al*. 2020). As eloquently articulated by Greenberg *et al*. (2020:2), healthcare staff ‘will be the heroes of the day, but we will need them for tomorrow’. Healthcare professionals must be provided with the supports necessary to perform to their full potential over the extended period of the response. Lessons learned from Italy, for example, point to staff suffering from work-related somatic sysmptoms, psychological burden and emotional stress (Barello *et al*. 2020).

Extreme demands on the healthcare service are likely to continue in various forms, especially considering the long-term impact of the physical and psychological implications of COVID-19 on the population. Furthermore, projections of a looming recession and the financial strain and economic adversity that will accompany this will have a significant impact on mental health and thus present further concerns for the pressure on mental health services (Mental Health Commission, 2011; Frasquilho *et al*. 2015).

## Child, adult and family perspectives

People availing of nursing inputs across the lifespan have been markedly affected. So far, only compulsory imposed restrictive social measures such as quarantine, physical distancing and school closures appear to be successful in interrupting virus transmission, at least temporarily, in many countries (Brooks *et al*. 2020; Leung *et al*. 2020). Children and adults have been confined to their homes with limited outdoor activities, and the loss of interaction with peers is likely to present a substantial negative impact on mental health and well-being (Dalton *et al*. 2020; Lades *et al*. 2020; Wang *et al*. 2020). Consequently, mental health issues and ability to cope with imposed social restrictions will be an ongoing challenge for families as well as the community in general.

Focusing on child and adolescent mental health, Dalton *et al*. (2020) argues that children are exposed to high levels of stress and anxiety whilst socially isolating with adults. Imposed restrictive social measures disrupt children’s lives and may result in negative physical, psychosocial and mental health consequences for them, their families and their communities (Brooks *et al*. 2020, Dalton *et al*. 2020; Wang *et al*. 2020). Children are faced with new challenges due to the lack of socialisation as their familiar norms are displaced. For example, they were not able to mix together within schools, creches or childminders, thus, hindering their ability to play with peers. Even when the resupmption of these services occur, it will be a very different experience with higher control measures related to inteactions.

Play is a right of every child according to the United Nations Convention of the Rights of the Child (Children’s Rights Alliance 2010), and the Department of Health in Ireland (2020) has launched ‘Let’s Play Ireland’ an initiative to encourage play during COVID-19. Unfortunately, COVID-19 has also added an additional burden of stress and anxiety to young adults preparing to sit state exams which determine their career choice. The uncertainty around these examinations and the changing from examination to calculated grades (Department of Education and Skills 2020) has been felt not only by the young adults themselves but also by their parents and the teaching faculty across the country. These students will indeed have a very different rite of passage; for instance, the further delayed publication of calculated results has disrupted the usual third level orientation and programme delivery modes. Whether and to what extent there will be a longer term effect is unclear, and thus, Dalton *et al*. (2020) caution against overlooking children’s needs. This sentiment holds true in providing children with accurate, understandable information but also listening to children and young peoples’ understanding in order to prevent anxiety.

Most children will be able to cope over time with the help of parents, family members, school staff and other trusted adults. Such influences have an essential role in helping children make sense of what they hear through honest, accurate information without overwhelming their fear or anxiety (European Centre for Disease Control 2020; Dalton *et al*. 2020). However, mental health face-to-face supports for children and young people are limited due to COVID-19 (Health Service Executive, 2020). Child and adolescent mental health service (CAMHS) community teams continue to engage with young people with mental health needs, as necessary. Consequently, many CAMHS teams are working in community liaison services providing one-to-one appointments by phone or video. The quality of the interaction between healthcare professionals and clients influences therapeutic relationships and everyday life experiences (Molin *et al*. 2016). While PPE is a key defence in COVID-19, the impact of N95 or FFP2 masks on client communication is uncertain (WHO, 2020). It has been noted, however, that face masks are not well tolerated by children or young people (European Centre for Disease Control, 2020). The potential effect of this on the therapeutic relationship is less studied; however, it is suffice to say that facemasks are anathema to routine mental healthcare practice.

## Older people, older person services and mental health impact

The impact of the COVID-19 restrictions has been more severe on older adults, both within the community environment, including access to healthcare services and disruption of routine home care, and within residential care. The reason is twofold. First, older people are at a higher risk of having underlying health conditions (cardiovascular disease, hypertension, chronic respiratory disease and diabetes), and are, therefore, more likely to develop more severe symptoms of COVID-19. Second, due to senescence, the immune system becomes less effective, and there is a compromised production of T and B lymphocytes which are responsible for generating antibodies in the body as well as the diminished function of mature lymphocytes (Montecino-Rodriguez *et al.* 2013, Weyend & Goronzy 2016). Other immune system challenges include difficulties in returning to pre-illness baseline, particularly where older people have frailty conditions.

Cocooning, as a public health intervention, has rarely been studied in older populations. Limited research which has examined cocooning is within populations of infants (pertussis and influenza), or in older people cocooning in relation to chronic pain (De Oliveria Lemos *et al*. 2019) and consumption cocooning (Zalega, 2018). What is known is that social isolation and loneliness, although distinct concepts, have a significant impact on physical health and mental health which includes anxiety, depression and cognitive decline as well as high blood pressure, obesity, accelerated sarcopenia and heart disease (NIH, 2019; Ward *et al*. 2019). Moreover, losing a sense of connection can change a person’s perception of the world and there is evidence to support social isolation and loneliness having greater or equivalent risk to mortality than smoking or obesity, respectively (NIH, 2019). Cocooning in Ireland appears to have a significant impact on older people’s mental health. For instance, data from ALONE’s helpline demonstrated that 62% of the recent 16 000 calls concerned mental health distress (ALONE, 2020). There is also the significant angst related to relatives not being able to visit (bi-directional), a fear of getting the virus and an uncertain end date to cocooning.

Within residential care facilities, the impact of COVID-19 has been substantial. A study from the United Kingdom examined five countries (Ireland, Belgium, France, Italy and Spain) and estimated that between 42% and 57% of total COVID-19 deaths occurred in care homes (Comas-Herrara *et al*. 2020). In addressing the ‘flattening of the curve’ theory, many countries’ responses to the pandemic have systematically focused on the acute care environment, particularly in the context of availability of ventilators. However, despite the obvious vulnerability of nursing homes, responses have predominantly been ‘too late, too little’ (Lloyd-Sherlock *et al*. 2020).

On 6th March 2020, Nursing Homes Ireland took the pre-emptive step of preventing visitors to private nursing homes. Although government commentaries at the time suggested this was unnecessary, later discussions pointed to this as a valuable decision. Despite this, clusters emerged within nursing homes and as of 10th May, there were 242 nursing home clusters reported to the HPSC within a further 150 clusters in residential settings, representing almost 52% of all clusters (HPSC, 2020).

The mental health impacts in these environments are likely to be significant. The lack of visitors to older people proved challenging for residents, families and staff. Coupled with this is the confusion in nursing homes related to a ‘new normal’. Residents were not able to engage in usual social interaction and familiar recreational activities with other residents; residents also feared catching the virus and dying, as many nursing home clusters have reported COVID-19 deaths. In addition, the prevalence of cognitive challenges (ie dementia) in nursing homes is high (Cahill, 2013), so understanding limitations on movement as well as being confused and frightened by unfamiliar PPE posed high distress levels. Again, the fear relatives have regarding the potential of their loved one catching the virus causes distress. This is compounded by issues such as negative media coverage. Moreover, traditional cultural appropriations of dying have also been impacted, for example, where only one sole relative is enabled to stay during the end of life period, limited touch (only via PPE) and transformed funeral servivces. In particular, witnessing a COVID-19 death is also very mentally stressful.

Within the context of staffing, nursing homes have struggled. First, staff are concerned regarding catching the virus in a vulnerable environment and potentially infecting their own families. Second, the impact of self-isolation after a positive test has put substantial pressure on Persons in Charge and Directors of Nursing related to continuity and quality of care, responding to the Health Information and Quality Authority (HIQA) reviews while concurrently limiting the spread. Moreover, all staff have developed long-term relationships with older residents, thus can also be impacted by their deaths.

## Societal impact and mental health

Ireland, like other countries, will have a significant challenge in responding to the impact of COVID-19 in relation to many areas. For example, due to the public health measures, Ireland’s routine commercial activities such as retail, tourism and hospitality as well as other non-essential services were closed. This translated to the government supporting impacted workers through COVID-19 payments with approximately 589 000 people receiving this payment in early May (Department of Employment Affairs and Social Protection, 2020).

Coupled with the fiscal impact on Ireland’s borrowing, there is a considerable stress related to economic recovery, in what is likely to be a further global recession. Unemployment has a significant impact on individuals’ mental health (Farré *et al*. 2018; Neubert *et al*. 2019).

In Ireland, approximately 38% of people have been negatively impacted by COVID-19 (CSO, 2020). This finding is supported in an Irish study (Lades *et al*. 2020) which found that social distancing may also pose significant mental health risks and markedly raise negative feelings. Moreover, COVID-19 information overload may increase stress.

There is also data indicating a rise in adverse health activities. There is a significant increase in junk food consumption, alcohol and tobacco consumption as well as sedentary activities such as time on the internet (CSO, 2020). The Central Statistics Office (2020) reports that overall life satisfaction has declined to 12.2% as opposed to 44.3% in 2018 while 76.9% report being either somewhat or very concerned regarding household stress related to confinement. In the context of domestic violence, Women’s Aid has expressed concerns regarding the impact of COVID-19 which concurs with a recent survey (CSO, 2020) demonstrating a drop in satisfaction with personal relationships from 60.0% in 2018 to 42.4% during COVID-19. While the effects of the pandemic are broad and the mental health contexts diverse, in our Supplementary material, we present the lived experience of one person availing of community mental health services to enrich our understanding of the perception of the mental health service response.

## Inpatient acute mental health: psychiatric units

While mental health nurses in Ireland work in a range of settings including outpatient, day services and community environments such as the person’s home (Cusack & Killoury, 2012), the acute inpatient environment, in the context of the pandemic, merits focus. In an acute care setting, social distancing may be a challenge for nurses caring for a person requiring one-to-one special observations. This may involve the registered nurse being within arm’s reach of the person. Also, social distancing cannot be maintained during rapid tranquillisation, particularly if physical restraint is required. Furthermore, in terms of discharge planning, it is not unusual to promote social and recreational activities that enhance interaction, engagement and provide social and cognitive stimulation. These are all evidence-based psychosocial interventions which help to reduce the frequency and intensity of non-cognitive symptoms of dementia (McGowan *et al*. 2019) in older adult care or affective disorders in general adult populations (Jordan-Halter, 2019). Routine mental health nursing activities of this type are now challenged. Intimate and other personal care often provided by nurses requires physical touch (Groven *et al*. 2017). Encouraging physical well-being, given that some people availing of mental health services experience a more significant burden of physical ill-health (Fogarty *et al*. 2020), is compromised. This is relevant as people living with severe and enduring mental health illness encounter greater vulnerability for metabolic syndrome (Health Service Executive, 2019) and may have less access to health-promoting activities. Infection control measures and access to specialist infection control advice are paramount. Li *et al*. (2019), however, identify challenges to this including insufficient numbers of specialist infection control nurses in mental health settings. They also report variable adherence to infection prevention and control measures secondary to illness factors and the physical environment, including in some cases, dormitories limiting opportunity for social distancing. Systems which promote close liaison with general medical services are particularly essential at this moment. Evidence that people attending mental health services have not enjoyed equivalent access as others to general medical services (Chiang *et al*. 2019; Fond *et al*. 2019; Sheridan, 2019) may be considered significant if replicated in the context of the COVID-19 pandemic. However, innovations have been evident in clinical settings, for instance, where staff members affix a photo of themselves to their PPE gowns. In these brief examples, it may be argued that essential mental health nursing services and by extension, people availing of these inputs, have been markedly affected by this pandemic. As an example, to demonstrate COVID-19 practice transformations and innovations, a perspective of a nurse working in acute inpatient care is provided in Supplementary material 2. This includes discussion of the nursing experience of the emergency amendments to the Mental Health Act.

## Conclusion

This paper has identified the actual and potential adverse health impact of COVID-19 within selected populations in Ireland. Mental health nursing has responded (and will continue to respond) to this challenge, and all nursing grades – clinical, management/leadership, education, policy and regulation must be commended alongside the work of our interdisciplinary and health systems’ colleagues. The COVID-19 emergency continues to develop, and both mental health professionals and people with personal experience of mental health illness are responding in new and innovative ways which may have seemed unimaginable a few short months ago. However, we have also focused on the role of other services and professionals including the interdisciplinary team, children’s nursing, older people’s care, health systems responses and individual level resilience and recovery-informed personal agency.

While the United Nations (United Nations, 2020: 14–16) have published a detailed policy brief calling attention to the mental health effects of COVID-19 and recommended actions, this paper emphasises issues related to an integrated care approach. This is typified through working with other colleagues in nursing (for instance, public health nursing, residential care nurses and children’s nursing) and within interdisciplinary teams to foster effective, person-centred partnerships with service users. It is argued that without a networked, integrated approach across traditional professional boundaries, the success of the nursing response to COVID-19 may be diminished. This is especially relevant, considering the potential of COVID-19 to become endemic and rooted in our communities for some time. Alongside this, careful consideration of the realities of working and living within the restrictions currently in place and the impact they exert on nurses, other healthcare staff and the people who avail of their care is necessary. In conclusion, it is intended that this contemporary account may inform practice and highlight issues of concern but with the essential dual purpose of cataloguing the response of nursing and people with lived experience to this unprecedented emergency.

## Supplementary material

For supplementary material accompanying this paper visit https://doi.org/10.1017/ipm.2020.105. click here to view supplementary material
