# Supplementary material for: The impact of COVID-19 on health systems, mental health and the potential for nursing
Source: Ir J Psychol Med. 2020 Sep 16:1–7. doi: 10.1017/ipm.2020.105 (PMC7596574; doi:10.1017/ipm.2020.105)
Supplement: Supplementary file 1 [file S0790966720001056sup.zip › S0790966720001056sup002.docx]

**Vignette 1 – A Perspective of a Person availing of Mental Health Services**

Minding my mental health throughout this pandemic is a challenge. As I have schizophrenia, the added complication of too much news, too much input and a lot of conspiracy theories to contend with add an extra layer of information for me to work through. But work through it I do; I take each question raised, analyse it and then dismiss it. I see the reality of the world that I am faced with as something I should deal with on a daily if not hourly basis. Structure is very important. I set an alarm each day and rise at that time. Though I am not working, due to COVID-19 I try to keep as disciplined a routine as possible. It is the comfort of normality that keeps me on the straight and narrow. I take my medication as prescribed. I try to limit my exposure to the constant stream of media regarding the pandemic and I do normal things – housework, walking, working in the garden, eating at regular times, the joy of cooking, talking to my wife and children and being with my dogs who are blissfully unaware of the situation we are in and welcome the extra contact, the cuddles on the couch, the walks within 2km of the house. When I was working, I worked as a schoolwarden (lollipop man) and I had to be at the same place every day at the same time. To be in work, we had to leave the house at 7:30 am every week day, my wife works as a lecturer, teaching in third level; two of my sons are both in secondary school, my eldest son is in art college in his final year. This pandemic has changed how we interact with the oustide world. My wife works remotely on the computer each day, critiquing her students work. My school going sons work via google classroom; my eldest son is submitting his final piece electronically. I had my first remote appointment - the phone rang a few days before the allotted time and it was my psychiatrist; we discussed my state of mind, whether any strange or confusing thoughts had entered it and the medication I was taking. Decisions were made after a number of searching questions. I was prepared - I had the email address of my chemist. All this happened whilst standing by our post-box outside my house. For a moment, I wondered what someone might have thought having overheard the conversation. A few days later I received a new appointment in the post. An effective solution to the difficulty of face to face contact. For the last number of years I have attended the national counselling service; this too has become remote with a weekly phone call. It’s a powerful way to do some work, a very welcome support and 45 minutes I look forward to every week. This is the new normal. After 8 weeks it is hard to imagine what kind of world we will return to when this pandemic is over. Being able to return to the things that we took for granted such as going to the cinema, seeing one’s psychiatrist, attending counselling, walking on the beach and sport. These things seem distant, but I do know they will return. I do not know when, but I have chosen not to worry about the things I do not have control over and concentrate on what I do have control on. I try to engender the relationships I have, keep in contact with my friends, my family and the professionals involved in my care as and when I need them. I welcome the opportunity to reflect on my life and not to take too seriously the challenges that this forced isolation brings. I know I am very lucky – I have a loving supportive family and environment, people who look out for me and clinicians that take my care and illness with the seriousness it should be taken with even if it is at a safe distance.
