# Supplementary material for: The impact of COVID-19 on health systems, mental health and the potential for nursing
Source: Ir J Psychol Med. 2020 Sep 16:1–7. doi: 10.1017/ipm.2020.105 (PMC7596574; doi:10.1017/ipm.2020.105)
Supplement: Supplementary file 1 [file S0790966720001056sup.zip › S0790966720001056sup001.docx]

**Vignette 2 – A Nurse’s Perspective**

COVID-19 has led to several changes within the mental health in-patient settings which are in line with standards across other disciplines such as the use of personal protective equipment; use of masks, gloves and wearing scrubs. Along with the recommended 2-meter social distancing measures being implemented to ensure safety of our staff in large multidisciplinary meetings, we have also used floor markings to remind staff and clients; especially clients who are cognitively impaired. A key skill which we all have honed within the last 8 weeks is our client education especially around hand hygiene and cough etiquette, with reminders and refreshers given regularly to those who need it. With the introduction of social distancing there has been more of an emphasis and effort placed on effective communication and interaction albeit with a heightened caution to ensure patient confidentiality, dignity and respect. Another enhancement due to COVID-19 is the introduction of the early warning score which is now utilised for all clients daily and temperature checks daily of staff. There has also been the re-purposing of a ward to become a COVID-19 positive quarantine unit to ensure isolation if a client is suspected to be COVID-19 positive. We are constantly adapting and growing, and we are rising to these challenges.

Changes to practice were required by the Emergency Measures in the Public Interest (Covid-19) Act 2020 which had implications for the Mental Health Act especially in relation to enabling the patient to present their case to the tribunal in writing as opposed to the traditional format of in person. With the focus of the deployment of resources aiming to prevent, minimise and limit people being infected with Covid-19, mental health facilities have been afforded the testing capacity for persons who are suspected of having Covid-19, however, additional focus has been required to support service users availing of online tribunals given poor concentration and other challenges arising from an altered mental state. During these unprecedented times additional personal protective measures have been taken during online tribunals and second opinions via video calls and phone calls. Overall, nursing care has continued with the addition of appropriate personal and protective equipment, social distancing measures in place and increased vigilance for infection amongst all patients and staff.
